# Construction and Application of Au NRs/4-MBA/PAM Ratiometric Surface-Enhanced Raman Scattering Substrate for Fish Veterinary Drug Residue Detection

**DOI:** 10.3390/nano14221774

**Published:** 2024-11-05

**Authors:** Jianxing Yu, Huiping Fu, Qing Gu

**Affiliations:** 1 Zhejiang Key Laboratory of Food Microbiology and Nutritional Health, School of Food Science and Biotechnology, Zhejiang Gongshang University, Hangzhou 310018, China; yujianxing@zjxu.edu.cn; 2 Jiaxing Key Laboratory of Molecular Recognition and Sensing, College of Biological, Chemical Sciences and Engineering, Jiaxing University, Jiaxing 314001, China; 3 College of Chemistry, Fuzhou University, Fuzhou 350116, China; fuhui7317@163.com

**Keywords:** Au nanorods, surface-enhanced Raman scattering, polyacrylamide, 4-mercaptobenzoic acid, veterinary drug residues

## Abstract

Surface-enhanced Raman scattering (SERS) is widely used for trace detection of substances, and the key to this technology lies in the preparation of the substrate material. In this study, a composite SERS material of Au NRs/4-MBA/PAM was constructed and characterized to better immobilize the reference molecule 4-mercaptobenzoic acid (4-MBA). Electron transmission microscopy results demonstrated that the PAM film helps Au NRs to pack closely, enhancing the stability of the material structure and reducing the interference of external environmental factors on the response of 4-MBA, thus improving the accuracy of quantitative determination. Comparative experimental results with the Au NRs/4-MBA substrate showed that the relative standard deviations (RSDs) of the detection results for MG on different batches of Au NRs/4-MBA/PAM were less than 8.0%, and the RSDs of different points on the same material were less than 10.0%, indicating that the Au NRs/4-MBA/PAM has higher uniformity, better reproducibility, and higher sensitivity in detecting malachite green (MG). Applying this material in the recovery determination of fish extract showed that the recovery rates of MG were between 75.60% and 83.24%. Therefore, the Au NRs/4-MBA/PAM substrate can accurately detect and quantify veterinary drug residue in complex matrices such as food tissue.

## 1. Introduction

Traditional Raman spectroscopy refers to the inelastic scattering between monochromatic light and molecular substances, where both the direction and energy of the light are altered after scattering. This method is characterized by a small scattering cross-section and weak signals [1,2]. Surface-enhanced Raman scattering (SERS) provides an exceptional boost to the Raman signal intensity, ranging from 10^6^ to 10^14^ times. This allows for the detection of individual molecules with unprecedented sensitivity. This capability addresses the limitations of conventional Raman spectroscopy, which is typically characterized by lower sensitivity [3,4]. SERS primarily utilizes the local surface plasmon resonance (LSPR) of nanomaterials to enhance Raman scattering. LSPR is a physical optical phenomenon that occurs within semiconductors and on the surface of metal particles with free electron gas. When incident light resonates with the free electron gas on the surface of metal particles, it generates a surface plasmon resonance effect, resulting in oscillating molecules that produce a tremendous amount of energy [5,6]. SERS is a powerful tool for the rapid and precise identification of trace substances, and it has broad applications across various domains, including pollutant surveillance [7], food safety [8], pharmaceutical analysis [9], explosive analysis [10], and biological sample detection [11].

The intensity of SERS signals is influenced by external environmental factors, the stability of the equipment, and the consistency of the substrate, among others. Enhancing the quantitative accuracy and reproducibility of SERS technology has been a hot research topic in recent years. The key to SERS technology lies in the preparation of the substrate; it is necessary to strictly control the structure of the nanomaterials to improve reproducibility and achieve a highly uniform substrate. Various nanostructure fabrication methods have been developed, such as electron-beam patterning [12], nanosphere patterning [13], and nanocontact printing [14]. Fiber-optic SERS sensors enable remote, in situ, and highly sensitive detection [15,16,17]. However, these methods are often costly, require expensive equipment, or are unstable over long periods, limiting their widespread employment for practice. To mitigate these concerns, specialists have introduced internal standards to correct for Raman intensity variations caused by changes in the testing environment, substrate non-uniformity, and laser intensity fluctuations, thereby advancing the fidelity of quantitative determinations and enhancing the credibility of the results [18].

One of the earliest methods used for quantitative analysis is the external addition of internal standards. By adding a certain amount of stable internal standard substance that does not interfere with the analyte to the sample and then measuring the comparative peak intensity of the target molecule versus the reference one, quantitative detection and analysis can be achieved [19]. Although the operation is simple, the internal standard molecules may compete for adsorption with the target molecules and may be affected by the external environment, leading to intensity changes [20]. The embedded internal standard method was developed to reduce this impact. This method usually embeds Raman probe molecules with dual functional groups in the core-shell nanostructure substrate, such as 4-Mercaptobenzoic acid (4-MBA) [21], DNA [22], and 1,4-Benzenedithiol (1,4-BDT) [23]. These molecules serve both as internal standard molecules and as linkers to form the shell. Yet, this demand imposes certain limitations on the selectivity spectrum of internal standard molecules. Bin Ren et al. [24] proposed a new method of embedding dual-substrate molecules in the core-shell structure, in which a molecule is selected as the internal standard with a pronounced Raman signal, and the other acts as the skeleton to form the shell. This method expands the selectivity range of internal standard molecules, reduces the possibility of internal standard molecules participating in competitive adsorption, and improves the reproducibility and accuracy of quantitative results. Nevertheless, achieving their versatility and controlled synthesis is challenging due to the complexity of their structure [25]. The preparation conditions for core-shell structured materials are very demanding and time-consuming, and the thickness of the shell layer is difficult to control; therefore, it is difficult to obtain the ideal core-internal standard molecule-shell structure [26].

In the present study, an advanced method for the preparation of Au NRs/4-MBA/PAM composite materials was proposed. We used polyacrylamide (PAM) to fix the reference molecule 4-MBA, thereby enhancing the uniformity and sensitivity of the membrane substrate. This composite material, designed as a SERS substrate, facilitates the quantitative detection and analysis of malachite green (MG), utilizing 4-MBA as the reference molecule for calibration. We calculated the ratio of the analyte MG’s peak value to that of the 4-MBA reference peak, thereby achieving quantitative detection of MG. It can effectively reduce the analytical errors caused by the uneven preparation of SERS substrates, thereby improving the accuracy of detection. Encapsulating a layer of PAM film on the Au nanorods helps in the close packing of nanoparticles, not only reducing the possibility of rival adsorptive interactions between the internal reference molecules and the analytes but also protecting the internal reference molecules from the impact of environmental factors [27]. Compared to the Au NRs/4-MBA substrate, the Au NRs/4-MBA/PAM film substrate exhibits higher uniformity, better reproducibility, and higher sensitivity. Moreover, the substrate preparation method is simple, showing extensive potential for application.

## 2. Materials and Methods

### 2.1. Materials and Reagents

Chloroauric acid (HAuCl_4_, 99.95%), cetyltrimethylammonium bromide (CTAB, 99%), sodium oleate (NaOL, 99%), sodium borohydride (NaBH4, 99%), silver nitrate (AgNO_3_, 99.8%), ascorbic acid (99%), 4-MBA (99%), PAM (99%), hydrochloric acid (HCl, 12.1 M), and malachite green standard solution (MG solution, 1%) were sourced from Shanghai Guoyao Group Chemical Reagent Co., Ltd. (Shanghai, China). The ultra-pure H_2_O used in the experiment was prepared using a Millipore water system (Merck, MA, USA). The fish meat was purchased from a local market in Fuzhou.

### 2.2. Instruments

We employed the Tecnai G2-F20 (FEI, CA, USA) for transmission electron microanalysis, the InVia Reflex (Renishaw, Gloucestershire, UK) for confocal Raman spectral intensity analysis, a BS124S analytical balance (Sartorius, Goslar, Germany) for weighing substrates, and an HC-3018 rapid centrifuge (Zhongkezhongjia, China) for the centrifugation process.

### 2.3. Preparation of Au NRs

The synthesis of Au nanorods (Au NRs) was carried out using the seed-mediated growth method assisted by dual surfactants, as described by Murray et al. [22]. The synthesis process begins with the preparation of gold seeds. With rigorous stirring, a freshly prepared, chilled solution of NaBH_4_ (0.60 mL, 10 mM) is swiftly infused to a blend of aqueous mixture (5.0 mL) comprising 0.50 mM of HAuCl_4_ and 0.20 M of CTAB. After the infusion, intense stirring continued for two minutes, during which the mixture’s appearance changed from a bright yellow to a more subdued yellow-brown hue; then, the mixing process was stopped. The solution was maintained at 30 °C for 30 min for later use.

Next, the preparation of gold growth solution was started by mixing reagents CTAB (3.425 g) and NaOL (0.594 g) in ultra-pure H_2_O (100 mL) in a beaker, and the mixture was reacted at 50 °C in an aqueous bath until it became lucid and transparent. Upon cooling the solution down to 30 °C, a 4.0 mM AgNO_3_ solution (4.649 mL) was injected, and the beaker was maintained at 30 °C for 15 min. Following this, a 1.1 mM HAuCl_4_ solution (88 mL) was infused, which was mixed at a rate of 700 rpm for 90 min until it turned colorless. Thereafter, HCl (12.1 M) was added to adjust the pH value to 1.39, the stirring speed was reduced to 400 rpm, and stirring continued for another 15 min.

Finally, 1.965 mL of a 15.76 mM ascorbic acid solution was dispensed into the mixture, which was then stirred vigorously for 30 s. Subsequently, 150 μL of the previously prepared gold seed solution was introduced, and vigorous stirring was extended for 30 s. The blended solution was placed at 30 °C for 24 h; during this period, the Au nanorods were incubated.

### 2.4. Synthesis Method Study of Au NRs/4-MBA/PAM

Different concentrations of polyacrylamide (PAM): four centrifuge tubes with 1.0 mL of Au NRs each were prepared and subjected to centrifugal processing at 8000 rpm for 12 min. The liquid phase was dropped, and then the sediment was redissolved in ultra-pure H_2_O (0.50 mL) in each tube. Then, 0.50 mL of 2.0 μg/mL 4-MBA solution was introduced to the four tubes. The mixtures were softly mixed for three hours at ambient temperature. Then, the tubes were subjected to centrifugal processing at 6000 rpm for 15 min, the liquid phase was dropped, and the sediment was redissolved with 50.0 μL of ultrapure H_2_O. The centrifugation, liquid phase elimination, and dispersion operations were repeated once. The dispersed mixture was then added to four tubes containing 450.0 μL of PAM with mass fractions of 0.024%, 0.12%, 0.6%, and 1.5%, respectively, and gently mixed for 25 min before being set aside for use.

Different concentrations of the internal standard (4-MBA): four centrifuge tubes with 1.0 mL of Au NRs each were prepared and subjected to centrifugal processing at 8000 rpm for 12 min. The liquid phase was dropped; then, the sediment was redissolved in 0.5 mL of ultra-pure H_2_O in each tube. Subsequently, 0.50 mL of 1.0 μg/mL, 2.0 μg/mL, 4.0 μg/mL, and 6.0 μg/mL 4-MBA solutions were introduced to the respective tubes. The tubes were gently mixed for three hours at ambient temperature. Following this, the tubes were subjected to centrifugal processing at 6000 rpm for 15 min, the liquid phase was dropped, and the sediment was redissolved with 50.0 μL of ultra-pure H_2_O. The centrifugation, liquid phase elimination, and dispersion operations were repeated once. The dispersed mixture was then added to four tubes containing 450.0 μL of 0.12% PAM each and gently mixed for 25 min before being set aside for use.

Different concentrations of Au NRs: twelve centrifuge tubes with 1.0 mL of Au NRs each were prepared and subjected to centrifugal processing at 8000 rpm for 12 min. The liquid phase was dropped, and the sediment was redissolved in 0.50 mL of ultrapure H_2_O in each tube. Then, 0.50 mL of a 2.0 μg/mL 4-MBA solution was added to the respective tubes. The mixtures were gently mixed for three hours at ambient temperature. Next, the tubes were processed using centrifugation at 6000 rpm for 15 min; the liquid phase was dropped into each tube, and the sediment was redissolved with 50.0 μL of ultra-pure H_2_O. The centrifugation and liquid phase elimination operations were repeated once. The sediments from the twelve tubes were collected into one tube and diluted with water to 120.0 μL. Aliquots of 5.0 μL, 10.0 μL, 20.0 μL, 30.0 μL, and 40.0 μL of the resulting solution were placed into five new tubes, respectively, and diluted with water to 50.0 μL. Finally, the mixture was added to five tubes containing 450.0 μL of PAM each, resulting in Au NRs concentrations of 0.5, 1, 2, 3, and 4 times the original concentration, and gently mixed for 25 min before being set aside for use.

### 2.5. Characterization of Materials

The microstructures of the substrate samples were characterized using the instruments mentioned above. A measure of 1 mL of the prepared nanoparticle solution was dropped onto a 3 mm × 3 mm microgrid copper mesh (TEM specific, FEI, CA, USA). After placing it in a 60 °C oven for one hour, TEM testing was conducted.

### 2.6. Fish Sample Preparation

A measure of 5.0 g of fish, minced into small pieces, was measured in a centrifuge tube, to which 10.0 mL of acetonitrile was introduced. The tube was capped, vortexed to ensure thorough mixing, and then ultrasonicated for 15 min. The blend was processed in a centrifuge at 4000 rpm for five minutes. The upper liquid phase was pipetted into a different centrifuge tube, the pellet was re-extracted in another 10.0 mL of acetonitrile, and the mixture was processed using centrifugation at 6000 rpm for three minutes. The liquid layers from the two steps were mixed, and acetonitrile was infused to reach a total volume of 25.0 mL, yielding the extraction solvent.

Neutral alumina (1.20 g) and the extraction solvent (7.0 mL) were transferred into a 15 mL centrifuge tube and then vortexed to ensure solution homogeneity, followed by centrifugal processing at 6000 rpm for three minutes. Next, 5.0 mL of the upper layer liquid was precisely and carefully pipetted into a separate centrifuge tube. After the liquid was completely evaporated using nitrogen gas, the remaining residue was reconstituted in 1.0 mL of acetonitrile–H_2_O blend. After ten minutes of ultrasonication, the solution was sifted through a 0.45 µm organic phase filter, yielding the blank fish matrix extract. The MG standard solution was diluted and added to the fish matrix to achieve final concentrations of 2.50 μg/L, 30.0 μg/L, and 50.0 μg/L in the extract.

### 2.7. SERS Detection with Au NRs/4-MBA/PAM

A volume of 5.0 μL of the Au NRs/4-MBA/PAM solution was applied onto a pristine silicon wafer and was further dried at 37 °C for one hour to form the Au NRs/4-MBA/PAM film. Subsequently, 5.0 μL of the MG/MG fish matrix extract was added to the film and dried at 37 °C for an additional hour. Raman detection was performed using an InVia Reflex spectrometer.

## 3. Results and Discussions

### 3.1. Characterization of Au NRs

**Figure 1:**
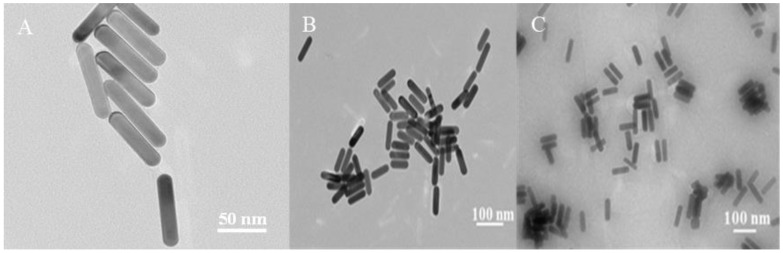
TEM characterization of (**A**) Au nanorods, (**B**) Au NRs/4-MBA substrate, and (**C**) Au NRs/4-MBA/PAM substrate.

Au nanorods exhibit two plasmonic absorption peaks, transverse and longitudinal, and the position of these peaks depends on the morphology, architecture, dimensions, and dielectric properties of the surrounding medium, given that these elements affect the electron charge distribution across the particle surface [28]. Figure 1A shows that the transmission electron micrograph illustrates the uniform size of the Au NRs.

### 3.2. Characterization of Au NRs/4-MBA/PAM

We conducted TEM characterization on the Au NRs, the Au NRs/4-MBA substrate, and the Au NRs/4-MBA/PAM film substrate. As shown in Figure 1, the Au NRs on the Au NRs/4-MBA/PAM film substrate are observed to be indistinct (Figure 1C), a phenomenon we attribute to the presence of the PAM film at the Au NRs interface. The PAM polymer may be sensitive to electron beams. The high-energy electron beam used in TEM may interact with PAM molecules, causing changes in the polymer structure, which can affect the clarity of the image.

### 3.3. Optimization of Synthesis Conditions

**Figure 2:**
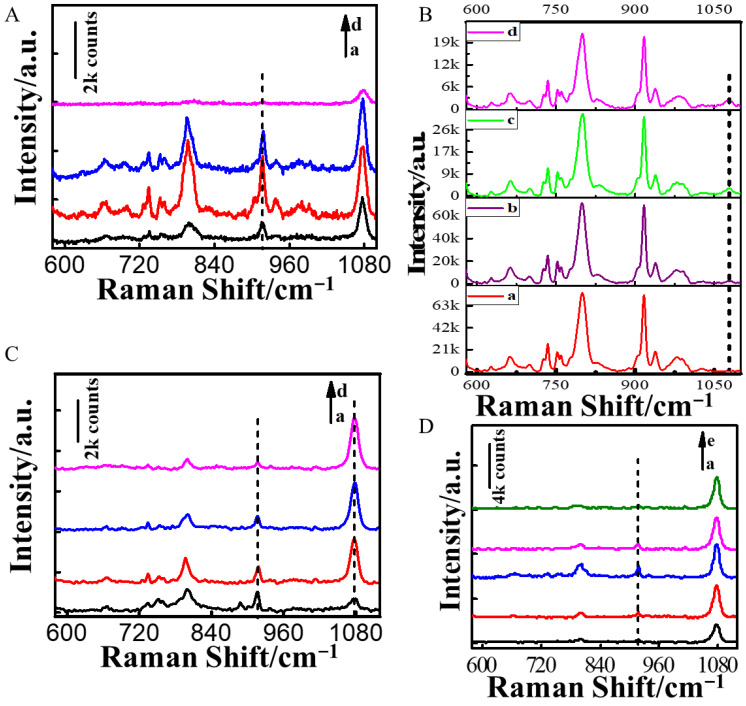
Comparison of SERS of substrates: (**A**) prepared with different concentrations of PAM: a–d represent 0.024%, 0.12%, 0.6%, and 1.5%; (**B**) prepared with different concentrations of 4-MBA when the MG concentration is 100.0 μg/L: a–d represent 1.0, 2.0, 4.0 and 6.0 μg/mL; (**C**) prepared with different concentrations of 4-MBA when the MG concentration is 5.0 μg/L: a–d represent 1.0, 2.0, 4.0 and 6.0 μg/mL; (**D**) prepared with different concentrations of Au NRs: a–e represent the concentrations of Au NRs at 0.5, 1, 2, 3, and 4 times the original concentration.

The performance of the Au NRs/4-MBA/PAM film substrate in SERS was contingent upon the thickness of the polyacrylamide film. We optimized the final concentration of the PAM solution. As depicted in Figure 2A, the SERS signal of MG rose with the increase in PAM concentration, which may be due to PAM promoting the close packing of Au NRs and facilitating the binding of the analyte to the nanoparticles. However, when the mass fraction of the PAM exceeded 0.12%, the SERS signal of MG weakened as the concentration of PAM increased, possibly because the resulting film was too thick, leading to the weakening of the SERS signal. Therefore, a mass fraction of 0.12% PAM was chosen for the preparation of the Au NRs/4-MBA/PAM film substrate in subsequent experiments.

The Raman maximum intensity at 1078 cm^−1^ was due to the C–C breathing mode oscillation within the aromatic ring of the internal standard molecule 4-MBA [29,30]. The observed maximum intensity served as a benchmark for assessing the substrate’s SERS efficiency and fine-tuning the 4-MBA concentration. SERS spectra were tested under two MG concentration conditions of 5.0 μg/mL and 100.0 μg/mL. As illustrated in Figure 2B, under the 100.0 μg/L concentration condition of the MG, the Raman intensity value at 1078 cm^−1^ exhibited a decline with the reduction in the reference molecule (4-MBA) concentration. When the 4-MBA molecule concentration was less than 1.0 μg/mL, the peak at 1078 cm^−1^ disappeared. The concentration of 4-MBA needed to be greater than 1.0 μg/mL to maximize the linear detection range for MG. Figure 2C showed that when the MG concentration was 5.0 μg/L, the Raman intensity of MG at 917 cm^−1^, as a result of the C–H bonds’ out-of-plane bending [31], decreased as the concentration of 4-MBA increased. After careful consideration, we chose a concentration of 4-MBA at 2.0 μg/mL as the optimal concentration for the preparation of the Au NRs/4-MBA/PAM film substrate in subsequent experiments.

We also optimized the concentration of Au NRs. Figure 2D compared the MG Raman spectra detected by the Au NRs/4-MBA/PAM film substrates prepared using varying concentrations of Au NRs. The figure clearly showed that as the Au NR concentration gradually increased, the intensity value of MG at 917 cm^−1^ also rose. When the Au NR concentration was twice the original concentration, the Raman intensity value was the highest. As the concentration further increased, the Raman intensity at 917 cm^−1^ decreased, likely due to the aggregation of Au NRs at high concentrations, which could cause a decline in the Raman signal. Based on the above situation, the concentration of Au NRs in subsequent experiments was selected to be twice the original concentration.

### 3.4. Performance Evaluation of Au NRs/4-MBA/PAM Film

**Figure 3:**
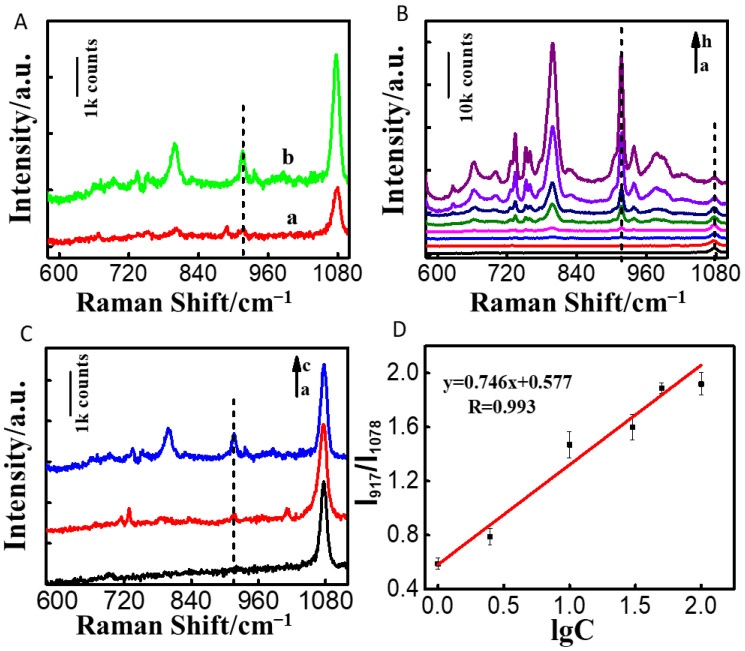
(**A**) Comparison of SERS signals for 1.0 μg/L of MG on (a) the Au NRs/4-MBA and (b) Au NRs/4-MBA/PAM; (**B**) comparison of SERS signals for varying levels of MG solutions with the Au NRs/4-MBA/PAM: a–h are 0, 0.75, 1.0, 2.50, 10.0, 30.0, 50.0, and 100.0 μg/L; (**C**) enlarged view of (**B**): a–c are 0, 0.75 and 1.0 μg/L; (**D**) the logarithmic linear calibration curve of I_917_/I_1078_ with MG concentrations spanning from 1.0 to 100.0 μg/L.

We analyzed the responsiveness of the Au NRs/4-MBA/PAM film substrate. By comparing the SERS signals of the Au NRs/4-MBA substrate with that of the Au NRs/4-MBA/PAM film substrate at a 1.0 μg/L concentration of MG, it was found that the representative peak of MG (917 cm^−1^) on the Au NRs/4-MBA/PAM film substrate was greater than that of the Au NRs/4-MBA substrate, indicating a stronger SERS signal (Figure 3A).

Polyacrylamide aided in the close packing of nanoparticles and facilitated the binding of the analyte to the nanoparticles. To further evaluate the sensitivity of the Au NRs/4-MBA/PAM film substrate, we measured the SERS signal intensity of MG at various concentrations on the substrate. As depicted in Figure 3B,C, under an MG concentration of 0.75 μg/L, the characteristic peak at 917 cm^−1^ could still be clearly identified. We used the representative peak of 4-MBA at 1078 cm^−1^ as the reference peak. As depicted in Figure 3D, the ratio of the height of the MG peak (917 cm^−1^) to the value of the internal standard 4-MBA (1078 cm^−1^) (I_917_/I_1078_) was linearly related to the log value of the MG concentration. This relationship had a linear range from 1.0 μg/L to 100.0 μg/L, achieving the calibration curve y = 0.746x + 0.577, an R coefficient of 0.993, and a limit of detection (LOD) of 0.75 μg/L (S/N > 3).

**Figure 4:**
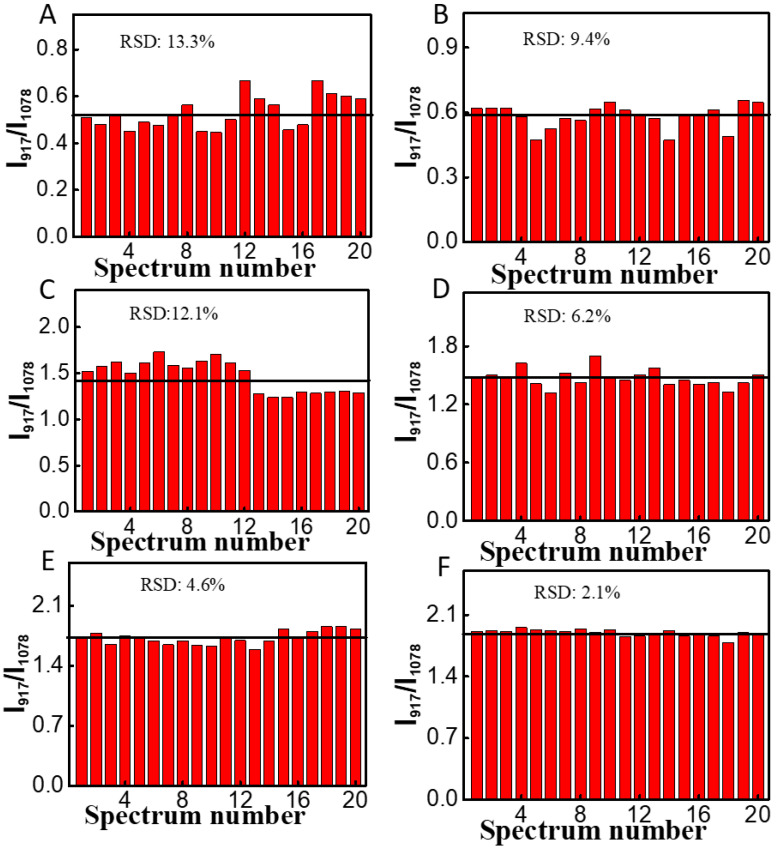
Comparison of measurement uniformity for MG at various concentrations between the Au NRs/4-MBA and Au NRs/4-MBA/PAM. Comparison of 1.0 μg/L of MG on (**A**) the Au NRs/4-MBA and (**B**) Au NRs/4-MBA/PAM substrate; comparison of 10.0 μg/L of MG on (**C**) the Au NRs/4-MBA and (**D**) Au NRs/4-MBA/PAM; comparison of 50.0 μg/L of MG on (**E**) the Au NRs/4-MBA and (**F**) Au NRs/4-MBA/PAM.

Additionally, we evaluated the uniformity of the Au NRs/4-MBA/PAM film substrate. We compared the uniformity of different concentrations of MG on the Au NRs/4-MBA substrate with that on the Au NRs/4-MBA/PAM film substrate. Twenty random points were selected on each substrate to calculate the relative standard deviation (RSD) of I_917_/I_1078_. When the concentrations of MG were 1.0, 10.0, and 50.0 μg/L, the uniformity of the Au NRs/4-MBA/PAM film substrate was better than that of the Au NRs/4-MBA substrate, with an RSD less than 10%, indicating good uniformity (as shown in Figure 4).

Next, we investigated the reproducibility of the substrates. We collected SERS spectra of 1.0 μg/L MG on three individual sets of Au NRs/4-MBA substrates and calculated the RSD of I_917_/I_1078_. Similarly, we collected SERS spectra of 1.0 μg/L MG on three different batches of Au NRs/4-MBA/PAM film substrates and calculated the RSD of I_917_/I_1078_. We compared the RSDs of the two types of substrates and performed SERS detection on the MG concentration of 10.0 and 50.0 μg/L. The results are shown in Table 1, where RSD (1), RSD (2), and RSD (3) represent the RSDs obtained at MG concentrations of 1.0, 10.0, and 50.0 μg/L, respectively. At concentrations of 1.0, 10.0, and 50.0 μg/L of MG, the reproducibility of the Au NRs/4-MBA/PAM film substrate was better than that of the Au NRs/4-MBA substrate, with RSDs less than 8.0%, indicating good reproducibility.

### 3.5. Influence of Fish Matrix on the SERS Detection of MG

**Figure 5:**
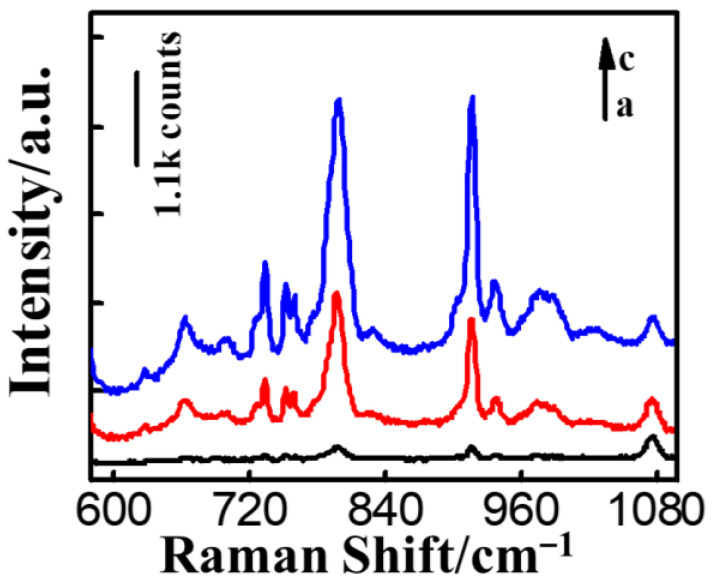
SERS spectra of the fish matrix with different concentrations of MG: a–c refer to 2.50, 30.0, and 50.0 μg/L.

In the preceding work, P. Kumar et al. crafted a flexible, ultrasensitive SERS sensor utilizing an Ag-coated structured PDMS substrate, showcasing remarkable sensitivity for MG detection at exceedingly low concentrations (10^−11^ M) [32]. This milestone sets a stringent benchmark for MG detection. To ascertain the applicability of our Au NRs/4-MBA/PAM film substrate for MG detection in complex samples, we introduced varying standard solutions of MG to a blank fish matrix, achieving MG concentrations of 2.50, 30.0, and 50.0 μg/L in the extract. Subsequent SERS analysis was conducted, with the spectral data presented in Figure 5. The calculated spiked recovery rates, as detailed in Table 2, ranged from 75.60% to 83.24% for MG. These recovery rates suggest the influence of matrix effects, where biomolecules such as proteins in the matrix may compete with MG for adsorption sites on the SERS substrate [33].

In our study, we specifically targeted the detection of MG at a concentration of 10^−10^ M within food samples using the Au NRs/4-MBA/PAM sensor. Our results indicate that the method we developed can effectively detect MG in complex matrices, such as those encountered in food samples.

## 4. Conclusions

The intensity of SERS signals is influenced by external environmental factors, fluctuations in laser intensity, and the uniformity of the substrate, making the quantitative accuracy and reproducibility of SERS technology significant challenges. In this study, we have designed and constructed an innovative Au NRs/4-MBA/PAM composite film for use as an internal standard-based SERS analytical probe. This novel substrate using 4-MBA as a reference molecule enables quantitative assessment of organic compounds, such as veterinary drug residues, through a ratio-metric analysis method. The PAM film coating on Au NRs not only facilitates the close packing of nanoparticles but also minimizes competitive adsorption and external environmental influences, thereby enhancing the accuracy of quantitative measurements. With a sensitivity range extending from 1.0 to 100.0 μg/L and a LOD of 0.75 μg/L, the Au NRs/4-MBA/PAM film substrate exhibits excellent uniformity, reproducibility, and sensitivity, as evidenced by the RSD values of less than 8.0% across batches and less than 10.0% within the same substrate. This work highlights the great potential of the Au NRs/4-MBA/PAM film substrate for preparing stable and uniform SERS substrates for detecting trace organic compounds in complex samples.

## Figures and Tables

**Table 1:** Comparison of RSD between different substrates.

Materials	RSD (1) %	RSD (2) %	RSD (3) %
Au NRs/4-MBA	11.8%	10.5%	4.3%
Au NRs/4-MBA/PAM	7.6%	5.8%	1.9%

**Table 2:** Recovery of different concentrations of MG in the fish matrix.

Sample	Added MG μg/L	Detected MG μg/L	Recovery %
Fish matrix	2.50	1.89	75.60
	30.00	24.70	82.33
	50.00	41.62	83.24

## Data Availability

Data are contained within the article.
